# Performance of the Emprint and Amica Microwave Ablation Systems in ex vivo Porcine Livers: Sphericity and Reproducibility Versus Size

**DOI:** 10.1007/s00270-020-02742-9

**Published:** 2021-01-18

**Authors:** P. Hendriks, W. E. M. Berkhout, C. I. Kaanen, J. H. Sluijter, I. J. Visser, J. J. van den Dobbelsteen, L. F. de Geus-Oei, A. G. Webb, M. C. Burgmans

**Affiliations:** 1 Department of Radiology, Leiden University Medical Center, P.O. Box 9600, RC Leiden, The Netherlands; 2 Medical Instruments & Bio-Inspired Technology, Faculty of Mechanical Engineering, Delft University of Technology, Delft, The Netherlands; 3 Biomedical Photonic Imaging Group, University of Twente, Enschede, The Netherlands; 4 C. J. Gorter Center for High Field MRI, Department of Radiology, Leiden University Medical Center, Leiden, The Netherlands

**Keywords:** Microwave ablation, Amica, Emprint, Ablation volume, Sphericity, Variability

## Abstract

**Purpose:**

To investigate the performance of two microwave ablation (MWA) systems regarding ablation volume, ablation shape and variability.

**Materials and Methods:**

In this ex vivo study, the Emprint and Amica MWA systems were used to ablate porcine livers at 4 different settings of time and power (3 and 5 minutes at 60 and 80 Watt). In total, 48 ablations were analysed for ablation size and shape using Vitrea Advanced Visualization software after acquisition of a 7T MRI scan.

**Results:**

Emprint ablations were smaller (11,1 vs. 21,1 mL *p *< 0.001), more spherical (sphericity index of 0.89 vs. 0.59 *p *< 0.001) and showed less variability than Amica ablations. In both systems, longer ablation time and higher power resulted in significantly larger ablation volumes.

**Conclusion:**

Emprint ablations were more spherical, and the results showed a lower variability than those of Amica ablations. This comes at the price of smaller ablation volumes.

**Supplementary information:**

The online version contains supplementary material available at (10.1007/s00270-020-02742-9).

## Introduction

Thermal ablation has become a widely accepted treatment modality for liver malignancies. In both primary and secondary liver tumours, thermal ablation is an effective, less invasive alternative to surgical resection of small lesions [1, 2]. Radiofrequency ablation (RFA) has been the most widely used thermal ablation technique, but microwave ablation (MWA) has rapidly gained popularity in recent years [3, 4].

Instead of using an electrical current, MWA uses an electromagnetic field at high frequencies that cause dielectric hysteresis, which results in tissue heating [5, 6]. As a result, MWA is associated with higher temperatures, larger ablation zones in a shorter time, and a lower susceptibility to properties of the surrounding tissue, in comparison with RFA [3, 7]. Propagation through (cirrhotic) tissue with a high impedance and heat sink effects in ablations near intrahepatic vessels are therefore less of an issue [6, 7]. Moreover, MWA does not require grounding pads, which reduces the chance of skin burns [8].

Nevertheless, MWA has certain disadvantages. The shape of MWA ablation zones has been described as being elliptical rather than spherical, compared with RFA [5]. Also, the size and shape of the coagulation necrosis tend to be less predictable using MWA [5]. Yet, predictability is of great importance to achieve favourable outcomes.

Local recurrence is the most common adverse event after thermal ablation, but oncological outcomes comparable to surgical resection can be achieved with the use of advanced planning and navigation tools [9, 10]. Highly sophisticated navigation software and robot assistance are now at the hand of interventional radiologists to optimize planning and guide needle placement [11, 12]. These tools make use of modelling techniques for which predictability of ablation shape and volume is a prerequisite. Ablation systems have predefined algorithms to predict the size and shape of the ablation and manufacturers provide reference values for ablations at different settings. In practice, however, these theoretical reference values deviate from actual dimensions of the coagulated tissue [13]. These deviations and lack of predictability currently hamper optimal use of treatment planning tools.

New microwave systems have been introduced trying to produce more spherical ablations and to overcome the issue of unpredictability. The Emprint ablation system (Covidien/Medtronic, Minneapolis, USA) is a new-generation microwave system that uses so-called thermosphere technology to control the microwave field and length of the microwaves. This technique combines thermal control by a cooling system that runs to the tip of the antenna with field shape and wavelength control [14]. It is claimed by the vendor that this new technology allows for more spherical and more predictable ablations. Although retrospective clinical cohort studies provide moderate evidence to these claims, there is a lack of studies comparing this newer ablation system with older generation microwave systems in a controlled setting [13].

To investigate and compare the performances of the Emprint ablation system, we conducted an ex vivo study with standardized needle placement in non-perfused, healthy porcine livers. An ex vivo study protocol was used to limit the influence of factors unrelated to the design and technology of the MWA systems. In a clinical setting, the geometry of the coagulation necrosis area would also be influenced by factors such as (adjustments in) needle position, hemodynamics and heat sink, tumour heterogeneity and/or capsule, cirrhosis/fibrosis etcetera. The performance of the Emprint system was compared with the Amica microwave system (HS Hospital Service, Rome, Italy), as this system is widely used and has been studied extensively in both in vivo and ex vivo studies [13]. The purpose of this experimental study was to investigate and compare the performance of these two systems regarding sphericity, reproducibility and ablation size.

## Materials and Methods

In this ex vivo animal study, 25 porcine livers were used. The livers were obtained at the abattoir and immediately stored in 0.9% NaCl solution at 5 °C.

### Microwave Ablation Systems

The first system used was the Emprint Ablation System with a generator with a maximum of 100 W at a frequency of 2.45 GHz. The second system was an Amica system powered by a HS-Amica-Gen (AGN-H-1.0) generator with a maximum output of 140 W, also at a frequency of 2.45 GHz. Both systems use a perfusion cooled antenna and a flexible coaxial cable. The 150-mm 14- and 11-gauge antennas were used for Amica and Emprint, respectively. There was no involvement of both manufacturers in this study.

### Ablation Protocol

**Figure 1:**
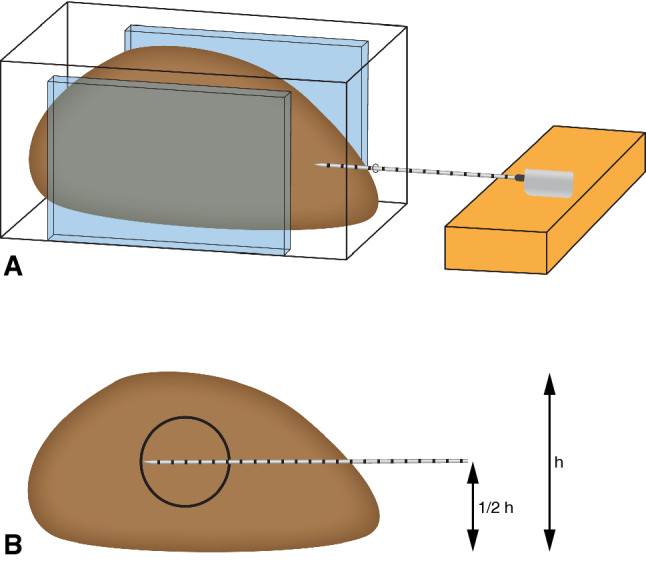
Experimental ablation set-up. The liver lobe is fixated within a plastic container with an antenna placed in the horizontal and vertical centre

Each porcine liver was divided into four parts, representing the four largest porcine liver lobes (left/right medial and lateral lobes). The lobar size had to exceed the expected ablation area by at least 5 mm on all sides (expected ablation sizes as derived from the manufacturer guidelines). Each liver lobe was positioned in a plastic box, fixated by placing additional plastic bars for an upright position, as shown in the schematic representation in Figure 1A.

A horizontal MWA antenna insertion point was chosen at half the height and width of the liver lobe, with a minimal insertion of 60 mm. The antenna positioning is shown in Figure 1B. A stable position of the antenna was ensured by fixation of the handle bar during ablation.

For both systems, ablations were performed at 4 different settings; alternating between 3 and 5 minutes of ablation time at both 60 and 80 Watt. An ablation was considered suitable for analysis if the intended ablation time was completed successfully, the ablation did not extend to the surface of the liver and MRI images were free of metal artefacts.

A total of 69 ablations were performed, of which 21 were excluded due to MRI artefacts (*n *= 14), ablation zones that reached the liver surface (*n *= 6), or an error in the cooling system (*n *= 1). Finally, 48 successful ablations were available for analysis: 6 for each setting and for each system.

### Assessment of Ablation Size and Geometry

In order to obtain volumetric data on the ablation necrosis, magnetic resonance imaging (MRI) was performed on all ablated liver lobes using a 7 Tesla MRI system (Achieva, Philips Healthcare, Best, The Netherlands) with a quadrature transmit head coil and 32-channel receive coil (Nova Medical). 3D T1-weighted gradient echo sequences were used with isotropic voxels of 1 mm (repetition time (TR) 4.19 ms, echo time (TE) 1.97 ms, flip angle 7°, field-of-view 200 x 200 x 200 mm, data matrix 200 x 200 x 200, 78% elliptical k-space coverage, radiofrequency spoiling between successive excitations, SENSE factor 2 in left-right direction).

**Figure 2:**
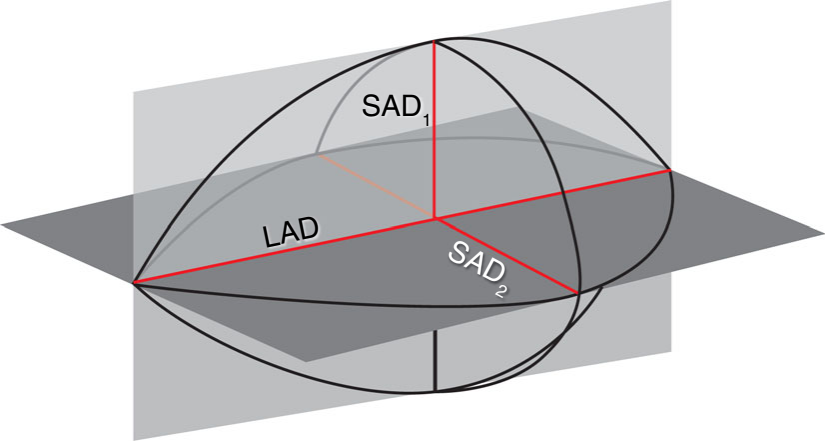
Short-axis diameters (SAD_1_ and SAD_2_) and long-axis diameter (LAD) of the ablation zone

Image processing was performed in Vitrea Advanced Visualization software (Vital Images, Minnetonka, USA) to evaluate the size and shape of each ablation. Ablation size was measured in millilitres (mL) and derived from the images using a semi-automated segmentation tool with adaptive thresholding. The ablation diameter was recorded in three axes, as shown in Figure 2: a long-axis diameter (LAD) in plane with the needle insertion axis and two orthogonal short-axis diameters (SAD). The sphericity index (SI) was defined as the ratio between those diameters $\frac{{\mathrm{SAD}}_{1}+{\mathrm{SAD}}_{2}}{2\mathrm{LAD}}$. An SI of 1 therefore denotes a perfectly spherical ablation, whereas a lower SI means that the ablation shape is more elliptical. Imaging parameters were acquired blinded from system and settings.

### Statistical Analysis

The performance of the two MWA systems was statistically analysed in terms of ablation volume and sphericity index. Statistical analyses were performed using IBM SPSS Statistics 25. Descriptive statistics were calculated for the outcomes of the different systems at the different settings, in terms of ablation time and power. The systems were compared using the unpaired T-test for normal distributed data or the Mann–Whitney U test for non-normally distributed data. Two-way analysis of variance (ANOVA) was conducted to test for differences in ablation volume and sphericity index between different ablation settings within one system. Three-way ANOVA was performed to test for differences in ablation volume and sphericity between both MWA systems in terms of time and power-settings. Normality of data was tested using skewness and kurtosis. Levene’s test was used to test for equal variances, and a 95% confidence interval was used.

## Results

**Figure 3:**
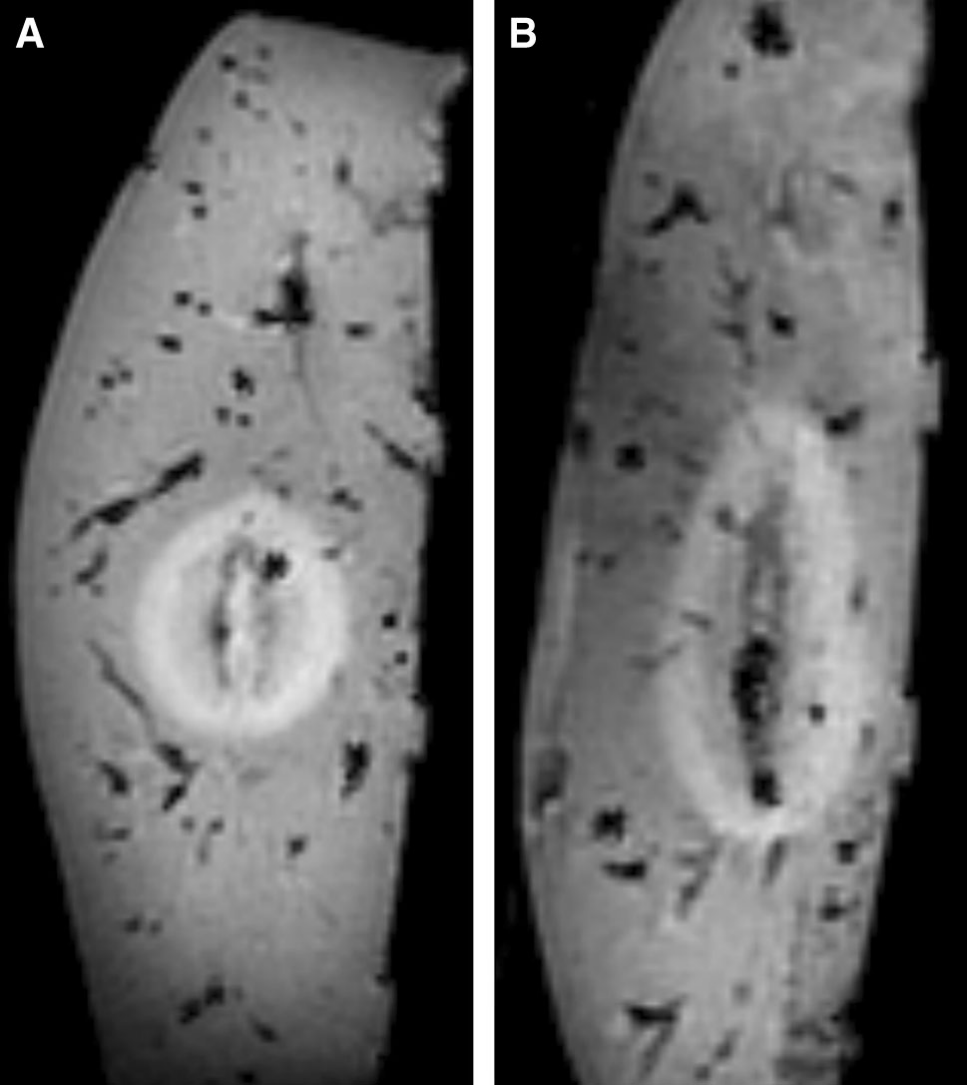
Sagittal MRI of ex vivo porcine livers after ablation. **A** Ablation zone after Emprint ablation of 3 minutes at 80 W. **B** Ablation zone of Amica ablation of 3 minutes at 80 W

**Figure 4:**
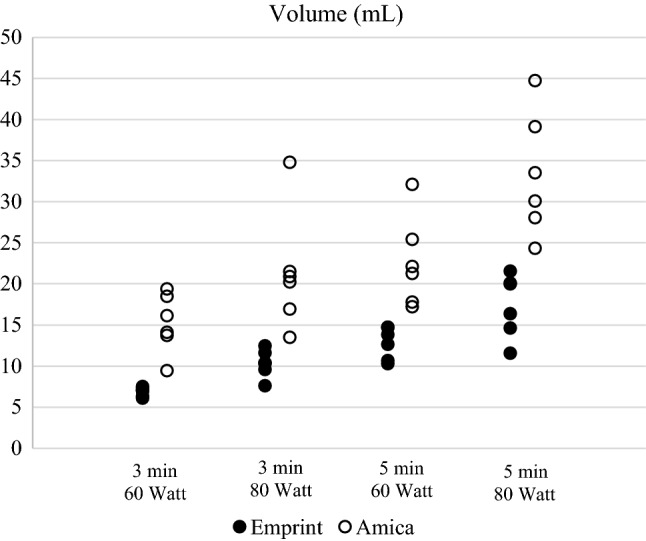
Each individual ablation volume per setting and system

An example of the post-ablation liver MRI can be found in Figure 3. Table 1 shows the median ablation volume for the 48 ablations. Amica ablations were significantly larger than Emprint ablation (*p *< 0.001), with a median ablation volume of 21.1 mL versus 11.1 mL. Figure 4 shows all individual ablation volumes per setting. For all settings, the range of ablation volumes was smaller for Emprint ablations compared to ablations produced with the Amica system (Table 1 and Figure 4).

**Table 1:** Ablation volume for each setting and system

Settings	Emprint (*n* = 24)		Amica (*n* = 24)	
	Median volume (mL)	(Range)	Median volume (mL)	(Range)
3 min, 60 W	7.0	(6.1–7.5)	15.1	(9.4–19.3)
3 min, 80 W	10.3	(7.6–12.4)	20.5	(13.4–34.7)
5 min, 60 W	13.2	(10.3–14.7)	21.7	(17.2–32.1)
5 min, 80 W	18.1	(11.5–21.5)	31.7	(24.3–44.7)
Total	11.1	(6.1–21.5)	21.1	(9.4–44.7)

Amica ablation volumes were significantly influenced by both ablation time (*p *= 0.001) and ablation power (*p *= 0.003). No interaction between those factors was revealed in two-way ANOVA analysis. The same results were found for Emprint ablation volume with p-values of *p*< 0.001 for both ablation time and power.

**Figure 5:**
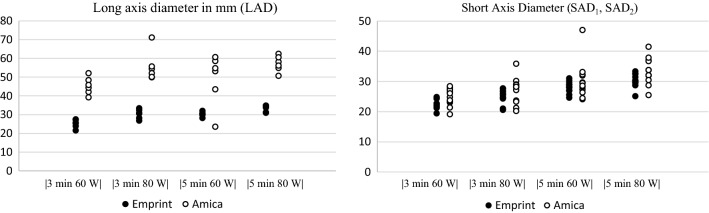
Ablation axis sizes for all individual ablations per setting and system: **A** long-axis diameter (LAD) was measured along the MWA antenna and **B** short-axis diameters (SAD_1_ and SAD_2_) were measured orthogonal to the LAD

Table 2 shows the median of the long-axis and short-axis diameters for different ablation settings. All individual measurements are plotted in Figure 5. Long-axis diameters were non-normally distributed. Mann–Whitney U statistics showed that the LAD was significantly larger for Amica ablations (*p *< 0.001). SADs did not significantly differ between the two systems. For all settings, there was a wider range of both LAD and SAD measurements for the Amica system compared to the Emprint system.

**Table 2:** Median long-axis and short-axis diameters for each setting and system

Settings	Emprint (*n* = 24)				Amica (*n* = 24)			
	Long-axis diameter (mm)		Short-ax-is diameter (mm)		Long-axis diameter (mm)		Short-axis diameter (mm)	
	Median	(Range)	Median	(Range)	Median	(Range)	Median	(Range)
3 min, 60 W	25.4	(21.4–27.3)	22.2	(19.4–24.8)	45.0	(39.0–51.9)	25.4	(19.1–28.4)
3 min, 80 W	30.6	(26.7–33.3)	25.5	(20.5–27.7)	53.1	(49.7–71.0)	28.1	(20.2–35.8)
5 min, 60 W	30.1	(28.0–31.9)	28.2	(24.6–31.0)	53.8	(23.4–60.5)	29.0	(24.1–47.0)
5 min, 80 W	33.9	(30.8–34.7)	30.1	(25.7–33.3)	57.0	(50.5–62.2)	32.9	(25.4–41.4)
Total	30.4	(21.4–34.7)	26.7	(19.4–33.3)	52.5	(39.0–71.0)	28.2	(19.1–47.0)

**Figure 6:**
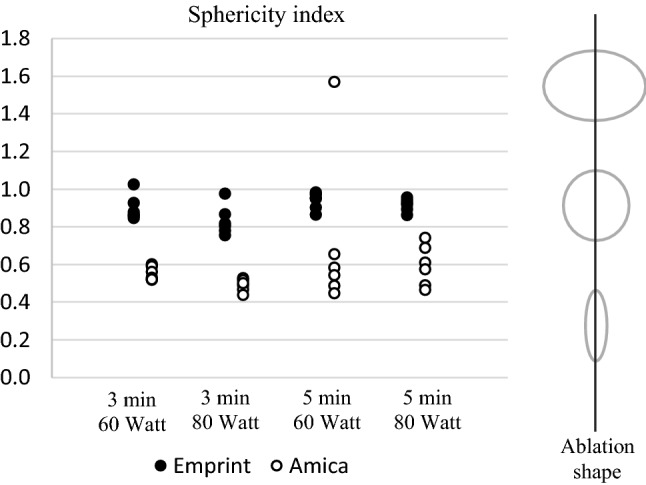
Sphericity index of the ablation zone for the Emprint and Amica system at different settings

The SI of Emprint was significantly higher (*p *< 0.001), as can be seen in Table 3. Figure 6 shows the SI of each measurement.

**Table 3:** Sphericity index for the Emprint and Amica system at different settings

Settings	Emprint (*n* = 24)		Amica (*n* = 24)	
	Mean sphericity index	(Range)	Mean sphericity index	(Range)
3 min, 60 W	0.90	(0.85–1.02)	0.55	(0.52–0.60)
3 min, 80 W	0.83	(0.75–0.97)	0.49	(0.44–0.52)
5 min, 60 W	0.93	(0.90–0.98)	0.71	(0.44–1.57)
5 min, 80 W	0.91	(0.86–0.95)	0.59	(0.46–0.74)
Total	0.89	(0.75–1.02)	0.59	(0.44–1.57)

In supplementary Figure 1, the ablation dimensions are plotted with respect to the manufacturers’ reference values.

## Discussion

In this study in ex vivo porcine livers, the Emprint ablation system created more reproducible ablation zones compared to the Amica system. The variation in repeated measurements for volume, LAD and SAD was smaller for the Emprint ablations. In addition, the Emprint ablation resulted in more consistent spherical ablations. As most liver malignancies tend to be rather spherical, this may be desirable in clinical practice. In larger tumours, the Amica system may offer an advantage. Especially due to a larger LAD (mean of 52.5 mm vs. 30.4 mm for the Emprint system), Amica ablations were significantly larger. The lower variability and higher sphericity of the Emprint system thus seem to come at the expense of ablations size.

Although no previous results are available for ex vivo Emprint ablations, our study is consistent with previously published studies with respect to Amica ablations. Amabile et al. performed Amica ablations in an in vivo porcine and ex vivo bovine study and found sphericity indices comparable to our study: 0.59 and 0.62 for 5-min ablation at 60 W and 80W, respectively, in the in vivo porcine model. This was 0.70 and 0.72 for the ex vivo bovine ablations at similar ablation parameters (compared to 0.71 and 0.59, respectively, in our study) [15]. Also, Hoffmann et al. reported similar results to ours for AMICA ablation volume and SAD (22.2 mL and 30.5 mm vs. 21.7 mL and 29.0 mm in our study with ablation settings of 5 min at 60 W) [16]. Our ex vivo findings also match reported clinical outcomes. Vogl et al. retrospectively analysed cross-sectional images of patients that underwent ablation with either an Amica or Emprint system [17]. Similar to our findings, they showed that Amica ablation volumes were larger (51.9 mm^3^ vs. 33.0 mm^3^) and less spherical (SI = 0.686 vs. SI = 0.865). In another study by Zaidi et al., including 53 patients treated with laparoscopic ablation with the Emprint system, ‘roundness indices’ were found to be 0.9, 1.0 and 1.1 in three different dimensions [18]. Head-to-head comparison of the two systems in a controlled environment has not been reported on previously.

The size and shape of ablation necrosis heavily depend on the propagation of heat through tissue. The complexity of heat conductivity can be reduced to the effects evaluated by the bioheat equation, which includes tissue properties, thermal conductivity, the rate at which heat is applied, and the heat loss (e.g. due to heat sink effect) [19]. Tissue properties are of high influence on the transmission of electromagnetic energy, due to their large effect on dielectric permittivity [19]. Porcine liver tissue has been shown to be suitable for simulating microwave energy distribution in healthy or tumourous liver tissue [20]. Earlier simulations with RFA revealed potential influences of fatty liver tissue on ablation volumes up to 27% and even 36% for cirrhotic liver tissue [21]. In theory, these rates should be lower for MWA than for RFA as MWA is less dependent on heat conductivity. Nevertheless, in practice the unpredictability of MWA systems has been an important limitation with earlier systems. Based on our study, this limitation has partly been overcome with the Emprint thermosphere technology. This makes it a more feasible system to use for precise treatment planning.

Emprint uses thermosphere technology that focuses on creating spherical ablation zones by thermal control, field control and wavelength control [14]. The antenna of Emprint is cooled all the way to the tip, which prevents undesired heating of surrounding tissue and aids in maintenance of a constant field and wavelength despite changing tissue (hydration properties) [22].

A newer Amica generator which has not been used in the current research offers the ability of pulsed ablations, striving for more spherical ablations as well. No results with respect to sphericity were found for this specific new system in the literature yet. However, in earlier research the effect of pulsed microwave ablation from another system was described as reaching similar ablation volumes at lower power, with limited differences in ablation shape when compared to non-pulsed MWA [23].

Despite the ex vivo character of this study, we chose to obtain our primary volumetric parameters by imaging analysis rather than histological analysis. In this way, we were able to perform accurate volumetric calculations and determine dimensions in a uniform way without risks of tissue deformation during sectioning.

There are several limitations of this study. First of all, ablations were performed in unperfused healthy porcine livers. The performance of the ablation systems used in this study may be different in clinical practice. Secondly, two different systems were used with each their own specifications. Therefore, a head-to-head comparison is not applicable to the full extent, i.e. no similar ablation size and volume were expected at similar settings of both systems. For both systems, different needle diameters are available. In this study, only 1 needle diameter was used for each system (14- and 11-gauge antennas were used for Amica and Emprint, respectively). Lastly, only two ablation systems were compared at a limited number of settings. In practice, more combinations in ablation time and power are expected to be used.

In conclusion, the Emprint system with thermosphere technology allows thermal ablation with greater reproducibility and more spherical ablations compared to the Amica system, in this ex vivo porcine study. This comes at the expense of smaller ablation volumes.

## Supplementary information

Experimental ablation dimensions compared to the reference values as provided by the manufacturers (JPG 258 kb)
