# Supplementary figures and images for: Performance of the Emprint and Amica Microwave Ablation Systems in ex vivo Porcine Livers: Sphericity and Reproducibility Versus Size
Source: Cardiovasc Intervent Radiol. 2021 Jan 18;44(6):952–8. doi: 10.1007/s00270-020-02742-9 (PMC8172387; doi:10.1007/s00270-020-02742-9)

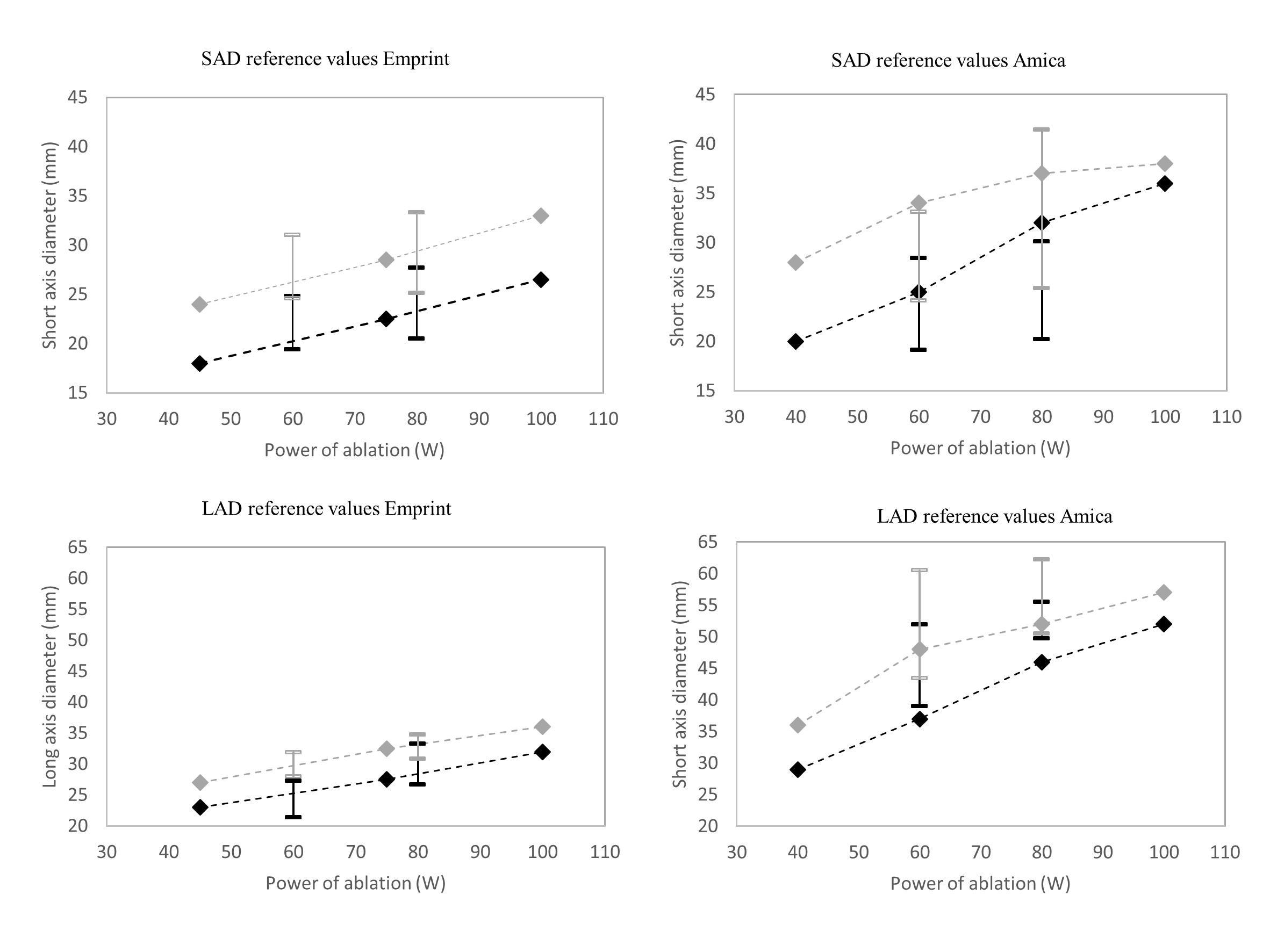

Supplement: Supplementary file 1 — Experimental ablation dimensions compared to the reference values as provided by the manufacturers (JPG 258 kb) [file 270_2020_2742_MOESM1_ESM.jpg]
